# Potential Impact of Frequent Testing for Hepatitis C Virus Among People Who Inject Drugs Toward Hepatitis C Elimination in the United States

**DOI:** 10.1093/cid/ciaf368

**Published:** 2025-07-15

**Authors:** Lin Zhu, Nathan W Furukawa, William W Thompson, Marissa B Reitsma, Liisa M Randall, Alice K Asher, Eduardo Valverde, Benjamin P Linas, Joshua A Salomon

**Affiliations:** Department of Health Policy, School of Medicine, Stanford University, Stanford, California, USA; National Center for HIV, Viral Hepatitis, STD, and TB Prevention, Centers for Disease Control and Prevention, Atlanta, Georgia, USA; National Center for HIV, Viral Hepatitis, STD, and TB Prevention, Centers for Disease Control and Prevention, Atlanta, Georgia, USA; Department of Health Policy, School of Medicine, Stanford University, Stanford, California, USA; Massachusetts Department of Public Health, Bureau of Infectious Disease and Laboratory Sciences, Boston, Massachusetts, USA; National Center for HIV, Viral Hepatitis, STD, and TB Prevention, Centers for Disease Control and Prevention, Atlanta, Georgia, USA; National Center for HIV, Viral Hepatitis, STD, and TB Prevention, Centers for Disease Control and Prevention, Atlanta, Georgia, USA; Section of Infectious Disease, Department of Medicine, Boston Medical Center, Boston, Massachusetts, USA; Department of Epidemiology, Boston University School of Public Health, Boston, Massachusetts, USA; Department of Health Policy, School of Medicine, Stanford University, Stanford, California, USA

**Keywords:** HCV, testing, elimination, PWID, network

## Abstract

**Background:**

Current hepatitis C virus (HCV) guidelines recommend testing at least once in a lifetime for all adults and periodic testing for people with ongoing risk factors. However, the testing frequency required for these populations including people who inject drugs (PWID) remains unknown.

**Methods:**

We developed a dynamic network model to simulate HCV transmission among PWID via sharing of injection equipment. We simulated different testing frequencies among PWID, paired with a treatment cascade of direct-acting antiviral therapy, and compared their impact on HCV infection prevalence, incidence, and HCV-related deaths over 10 years. We conducted sensitivity analyses on key parameters and assumptions.

**Results:**

In the main analysis, testing on average once per 6 months reduced HCV infection prevalence, incidence, and HCV-related deaths by up to 45%, 37%, and 42%, respectively. In the sensitivity analyses, increased coverage (access and utilization) had the greatest impact on all three outcomes, and reinfection risk after cure had a substantial impact on incidence. Lower transmission risk, higher cessation rate of injection, and longer partnership duration decreased the 3 outcomes with or without HCV testing.

**Conclusions:**

Frequent HCV testing with treatment had a substantial impact on hepatitis C elimination outcomes. However, it alone in the context of current continuum of care among PWID is unlikely to be sufficient to achieving elimination. Improving access to and utilization of hepatitis C care and harm-reduction services among PWID are crucial to achieving elimination.

## Teaser

Frequent hepatitis C virus testing will contribute to hepatitis C elimination among people who inject drugs but is unlikely to achieve elimination in the absence of comprehensive strategies including expanding hepatitis C care access and utilization and harm-reduction services.

**(See the Editorial Commentary by Grebely on pages e47–9.)**

The United States adopted the hepatitis C elimination goal to reduce new hepatitis C virus (HCV) infections by 90% and HCV-related deaths by 65% by 2030 [1]. The elimination of hepatitis C involves reducing prevalence and preventing transmission [2]. Highly efficacious and well tolerated direct-acting antiviral (DAA) treatments can not only cure HCV infection and reduce prevalence [3] but also potentially interrupt HCV transmission, as exemplified in “treatment as prevention” [4]. HCV treatment therefore constitutes a critical component of the elimination strategy. However, the impact of DAA treatment depends on people infected being identified and engaged in hepatitis C care.

Due in part to asymptomatic infection, approximately one third of people chronically infected with HCV in the United States are unaware of their infection [5]. In 2020, the national HCV screening guidelines were updated to recommend screening at least once in a lifetime for all adults [6, 7]. The impact of universal screening on populations at increased risk, including people who inject drugs (PWID), may be limited due to lack of access to and utilization of clinical care [2, 8]. The ongoing risk of reinfection due to sharing of injection equipment can further reduce the benefits of one-time screening and treatment among PWID [9]. Current guidance recognizes the need for “ongoing periodic screening” among certain individuals including PWID but provides no guidance on the frequency of repeated testing [10].

Mathematical models have been used to evaluate hepatitis C elimination strategies among PWID [11]. A number of studies have evaluated the annual treatment rate required to achieve elimination [4, 12–14]. Cipriano et al assessed the health and economic impacts of 1-time and repeated HCV testing in opioid treatment programs in the pre-DAA era [15]. Further studies are needed to identify optimal testing intervals among PWID in the DAA era to inform clinical practice. Most prior studies have been based on deterministic compartmental models [11]. However, typical simplifications in these models that limit heterogeneity of the PWID population may result in more optimistic results. For example, models may assume that all PWID have the same probability of receiving testing and treatment, although in reality limited engagement with intervention programs among subsets of PWID may facilitate continued viral persistence and transmission within affected networks [16]. Additionally, an individual's infection and treatment history may affect future behavior [17], but approaches to track history are limited in compartmental models compared to individual-based models. Furthermore, HCV is mainly transmitted through the sharing of drug-injecting equipment among PWID [18], and the complex network of injection partnerships can impact HCV transmission [19–21]. In this study, we therefore use an agent-based network model to simulate heterogeneous injection partnerships and HCV transmission risks among PWID in different settings to evaluate the potential contributions of frequent HCV testing toward hepatitis C elimination.

## METHODS

### Analytic Overview

In this study we extended a previously developed network simulation model of injection-equipment-sharing partnerships and HCV transmission among PWID [22]. We used data from the Social Networks among Appalachian People (SNAP) study [23, 24], a longitudinal study of people who use drugs that collected information on demographics, drugs, and detailed dyad-level characteristics of injection partnerships, complemented by published literature on PWID in rural and urban US to inform model inputs and calibration. We assessed intervention outcomes in 2 settings with different transmission intensity and conducted sensitivity and scenario analyses on key parameters and assumptions. Table 1 presents model parameters, values, and sources.

**Table 1.** Input Parameters for Simulations Using the Dynamic Network Model of People Who Inject Drugs (PWID)

Parameters	Values	Reference
Injection drug use dynamics		
Monthly injecting initiation probability^a^	0.002	Calibration
Monthly injecting cessation probability^b^	0.014	Technical Appendix
Monthly injecting relapse probability^b^	0.033	Technical Appendix
Probability of permanent cessation of injecting^b^	0.13	Technical Appendix
Mortality		
SMR for former PWID^c^	1.8	Technical Appendix
SMR for current PWID^c^	6.1	Technical Appendix
Additional monthly mortality rate for F4^d^	0.0001	Technical Appendix
Additional monthly mortality rate for decompensated^d^	0.0128	Technical Appendix
Post-SVR mortality multiplier for F4 and decompensated^d^	0.29	Technical Appendix
Network simulations		
Initial number of PWID	1000	[22], Supplementary Table 1, and assumption
Initial number of people who ceased injection	373	
Baseline HCV antibody positivity (%)	59	
Sparse network mean degree (average number of injection equipment-sharing partners)	1.43	
Dense network mean degree	3	
Ratio of mean degree between HCV antibody (+) and (−) PWID	1.73	
Proportion of population with no partners (isolates) (%)	37	
HCV sero-discordant partnerships (%)	29	
Transitivity (Geometrically Weighted Edgewise Shared Partnerships density^e^)	0.28	
Average equipment-sharing partner duration (y)^f^	3	[25], SNAP, and assumption
HCV infection and progression		
Monthly transmission probability in discordant partnerships	0.031	calibration
Spontaneous clearance of acute infection (%)	25	[26]
Probability of HCV seroconversion after different periods of time following acute infection^g^		Technical Appendix
1 m	0.05	
2 m	0.5	
3 m	0.9	
4 m and after	0.99	
Monthly fibrosis progression probabilities		[27]
F0–F1	0.008877	
F1–F2	0.00681	
F2–F3	0.0097026	
F3–F4	0.0096201	
F4-decompensated	0.0097026	
HCV testing and DAA treatment		
Testing coverage (%)^h^	60	[8, 28, 29]
HCV antibody test specificity^i^	0.99945	[30]
HCV antibody test sensitivity^i^	1	[30]
HCV RNA test specificity^i^	0.999985	[30]
HCV RNA testing sensitivity^i^	1	[30]
Linkage to care (ie, scheduled follow-up doctor appointment) (%)	46.9	[31–33]
Average interval between positive test result and treatment initiation (m)	3	[34]
Treatment initiation among those linked to care (%)^j^	92	[35]
Treatment completion among those who initiate (%)	96	[36, 37]
Cure rate (SVR among those who complete treatment) (%)	94	[37, 38]
Relative risk of reinfection after cure (compared to risk of primary infection)^k^	0.34	[9, 17, 39, 40]

Abbreviations: DAA, direct-acting antiviral; HCV, hepatitis C virus; MOUD, medications for opioid use disorder; PWID, people who inject drugs; SMR, standardized mortality ratio; STERGM, separable temporal exponential random graph models; SVR, sustained virologic response.

^a^Number of new PWID per month was calculated by multiplying this rate and initial population size (1000). Initiation rate was calibrated to keep population size of current PWID stable.

^b^We used Kaplan–Meier estimators of time to cessation and relapse (conditional on cessation) in the ALIVE study to estimate the 3 parameters (details in Technical Appendix).

^c^These are overall SMRs for all ages. We generated age-dependent SMRs by fitting a regression to multiple SMR and age group points described in detail in Technical Appendix.

^d^Calculation is described in detail in Technical Appendix.

^e^Proportion of 2 stars (2 nodes connected to a common node) that are closed (formed triangle).

^f^Analysis from the SNAP data shows average duration of “acquaintance” between injection partners was 10 y; in the cited study, the reported median duration of “know each other” was 10 m, and duration of “injected with each other” was 4.5 m. We assumed an average duration of 3 y in the main analysis.

^g^The assumptions are based on multiple studies on timing of HCV antibody development, details are summarized in the Technical Appendix.

^h^Testing coverage is defined as the percentage of PWID that have access to and utilize regular HCV testing. We used %PWID ever tested for HCV in literature to approximate this parameter. According to the systematic review and meta-analysis, the value was 77%; and the NHBS 2018 reported 80%. Due to the large weight on urban areas and a few studies included having samples from harm-reduction programs, which could overestimate the value, we assumed a more conservative value of 60%, and added sensitivity analyses of 30% and 90%, spanning beyond 80%. We modeled reflex testing, that is, individuals first receive antibody testing and those who are antibody positive automatically receive RNA testing.

^i^Using mean value of the approved assays.

^j^Previously, many states have imposed strict eligibility criteria for Medicaid coverage of DAAs, for example, more advanced fibrosis stage, abstinence from substance use, which prevented PWID from treatment initiation even after visiting a provider. In recent years, most states have begun to eliminate these coverage restrictions, so we used a common treatment initiation rate among those linked to care, and used linkage to care to reflect the remaining barriers for treatment uptake.

^k^In the 2 cited meta-analyses, the rate of HCV reinfection after treatment was 6.2/100 person-years among people who recently injected drugs, and the gender-weighted rate of HCV primary infection among PWID was 18/100 person-years. The rate ratio of reinfection after treatment was thus estimated to be 6.2/18 = 0.34, which would translate to a relative risk equal to this value. We used this same value for reinfection during treatment, because the first citation reported that 80% of people continued using drugs during treatment, and the fourth citation reported that treatment reduced drug use but reported no change in sharing behavior.

### Network Simulation

We simulated the PWID population individually characterized by age, HCV infection status, liver fibrosis stage, and injection drug use status. Inputs were informed by estimates from SNAP data and published literature (Technical Appendix). The initial seroprevalence was 59% [22] and changed over time as a function of varying incidences.

We used exponential random graph models [41] to simulate injection partnerships among PWID, reproducing observed network characteristics of the average number of injecting partners per person (mean degree), proportion of PWID with no injecting partners (isolates), mean degree by HCV serostatus, HCV serostatus concordance, and transitivity (“partner of a partner is more likely to be a partner” phenomenon). We used estimates from SNAP data to define parameters for a lower-transmission rural (“sparse”) network and used published literature (Supplementary Table 1) to inform the mean degree value in a higher-transmission urban (“dense”) network. Although we did not explicitly simulate harm-reduction interventions in the main analysis, drug use behaviors implicitly reflect status quo levels of these interventions. For instance, coverage of syringe services can result in an individual having fewer sharing partners, which lowers the mean degree. The sparse network setting may therefore reflect dynamics in a less-connected PWID population, or a PWID population that has high coverage of syringe services.

**Figure 1.**
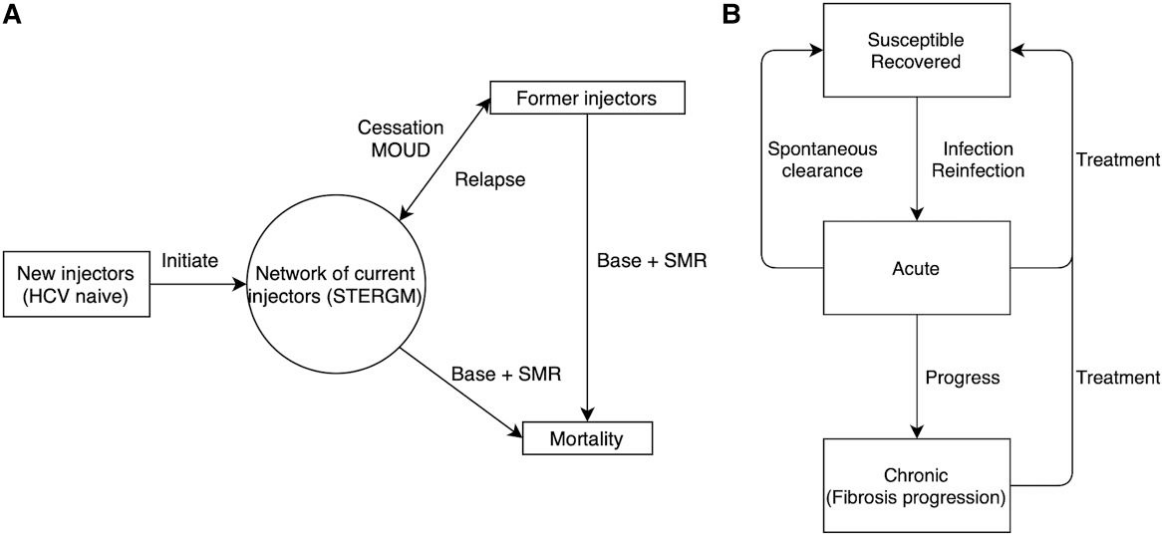
Model diagram: network simulation, population dynamics, and natural history of HCV infection. Panel *A* summarizes the network model, which consists of an active PWID network with partnership formation and dissolution simulated with STERGM, and population dynamics of initiation, cessation, and relapse of injection, and deaths. Panel *B* summarizes the natural history of HCV infection simulated in the model: after an acute HCV infection in a susceptible or recovered individual, there is a probability of spontaneous clearance before progressing into chronic infection, when fibrosis progression could happen. Infected individuals also have a probability of recovering through DAA treatment. Abbreviations: DAA, direct-acting antiviral; HCV, hepatitis C virus; MOUD, medications for opioid use disorder; PWID, people who inject drugs; SMR, standardized mortality ratio; STERGM, separable temporal exponential random graph models.

Building on our prior static network model [22], we used separable temporal exponential random graph models (STERGMs) with the Statnet package [42] to simulate the dynamic formation and dissolution of partnerships over time based on average partnership duration. We allowed HCV infection and injecting partnerships to evolve independently over time, while preserving other network characteristics including mean degree, isolates, and transitivity. Individuals can move into or out of the active injection network by initiation/cessation/relapse of injection drug use or deaths (Technical Appendix). The simulation of population dynamics is summarized in Figure 1*A*.

### Natural History of HCV Infection and Mortality

Individuals can acquire infection from their injection partners. The monthly transmission probability between HCV-discordant partners was calibrated with seroprevalence and incidence data from SNAP [22]. Based on findings from 2 meta-analyses reporting substantially lower reinfection incidence compared to primary infection [39, 40], and the observation of reduced injection drug use during and after DAA treatment [43], we assumed that the transmission probability after cure would be approximately two-thirds lower than the transmission probability for primary infection (calculations presented in Table 1, footnote k). People with recent infection may spontaneously clear the infection [26] (Figure 1*B*). People who develop chronic infection can have progressively worsening liver fibrosis [27]. Each PWID in the model is assigned an age-dependent base mortality rate, with excess mortality rates associated with injection drug use, HCV infection status, and fibrosis stage (Technical Appendix).

### Testing and Treatment Cascade

HCV testing in each PWID was determined by 2 parameters: coverage and testing frequency. The coverage parameter is defined as the percentage of PWID that have access to and utilize regular HCV testing, accounting for the empirical observation that not all PWID can be reached by HCV testing [28, 29]. The base case value was 60% (details in Table 1, footnote h). We simulated 3 HCV testing frequencies: every 2 years, every year, and every 6 months, implemented by varying the monthly testing probability (1/frequency) among those who can be reached by testing. We conducted an additional analysis examining the impact of testing every three months, which may be considered infeasible in most settings, but provides a reference upper bound.

Tested individuals first receive HCV antibody testing and those who are positive automatically receive RNA testing. For individuals who are diagnosed, we modeled a treatment cascade including linkage to care (visiting a prescribing provider), DAA treatment initiation, completion, and sustained virologic response (SVR). With the base-case cascade values (Table 1), the treatment uptake (proportion of diagnosed initiating treatment) was 43%, consistent with the average in empirical studies [29, 44].

### Sensitivity and Scenario Analyses

We performed 1-way sensitivity analyses on parameters governing testing coverage, linkage to care, monthly transmission probability, and average partnership duration, by varying their values to be 50% higher or 50% lower than base-case values in the main analysis. We altered the value of linkage to care to represent potential changes in treatment uptake. We also conducted a sensitivity analysis on the relative risk of reinfection after cure compared to risk of primary infection, with values of zero representing no reinfection after cure and one representing equal infection risk before and after treatment. We performed a scenario analysis in which we reduced the monthly transmission probability by 25% (which is roughly half of the reported effect size of syringe services programs [SSPs], on transmission [45]) and increased injection cessation rate by 50%, to mimic a scenario with ongoing harm-reduction services including SSPs and medications for opioid use disorder (MOUD). To test the robustness of the estimated benefits of testing to less favorable assumptions about test performance and spontaneous clearance, we conducted additional sensitivity analyses using lower bound values for sensitivity (96.7% [46]) and specificity (99%, expert opinion) of antibody testing, and higher rate of spontaneous clearance of 50% [47] (compared to the base case of 25% [26]).

### Simulations, Outcomes, and Analysis

We ran the simulation in monthly time steps. To allow for stochastic variation across simulations, we ran 500 iterations for each testing frequency, sensitivity analysis, and scenario analysis scenario and report the mean across the 500 iterations. The time horizon for each simulation was 10 years. Primary outcomes included (1) average HCV infection prevalence, computed as cumulative person-years of HCV infection divided by cumulative person-years lived in the simulated PWID population; (2) cumulative HCV infection incidence, computed as cumulative number of HCV infections divided by cumulative person-years lived by susceptible PWID; and (3) cumulative number of HCV-related deaths.

## RESULTS

### Main Analysis

**Figure 2.**
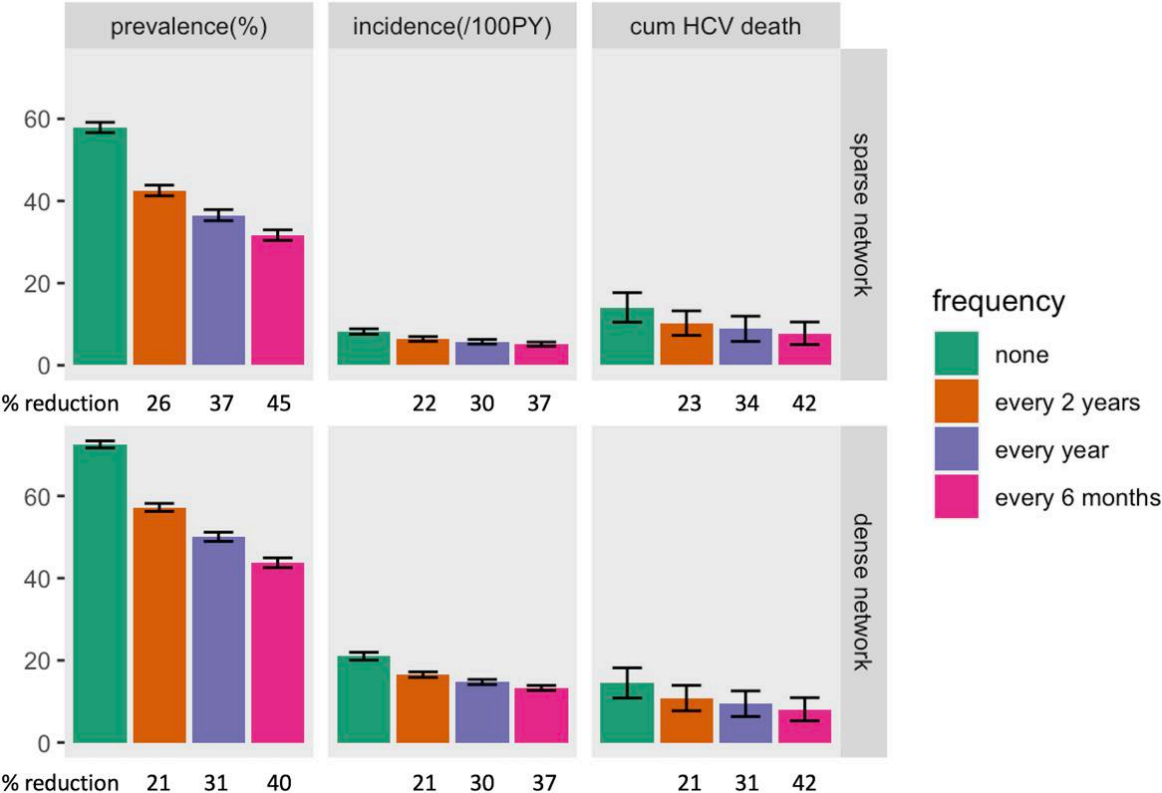
Main analysis: HCV infection prevalence, incidence, and cumulative HCV-related deaths over 10 y in PWID with different HCV testing frequencies. The top panels show results for sparse PWID network simulations (average number of injection partners = 1.43), and the bottom panels show results for dense PWID network simulations (average number of injection partners = 3). Three outcome measures are presented, each in a column: HCV infection prevalence (%), HCV infection incidence (per 100 person-years), and cumulative number of HCV-related deaths over 10 years. In each panel, the different colors represent different screening frequencies: none, every 2 years, every year, and every 6 months, as described in the legend. Height of each bar shows the mean of the outcome values (over 500 iterations of simulations), and the error bar shows the standard deviation. The number below each bar shows relative/percentage reductions compared to no screening. Abbreviations: HCV, hepatitis C virus; PWID, people who inject drugs; PY, person-years.

The 2 network settings exhibited different levels of HCV transmission: as shown in Figure 2, the absence of HCV testing and treatment over the 10-year simulation period resulted in an average prevalence of 58% and an incidence rate of 8/100 person-years in the sparse network, and 73% and 21/100 person-years, respectively, in the dense network. The cumulative number of HCV-related deaths was consistent in both settings, totaling 14 over 10 years. Notably, the cumulative deaths attributable to injection drug use were 9 times greater than HCV-related deaths.

HCV testing and treatment reduced prevalence, incidence, and HCV-related deaths in both settings, with the reductions being positively correlated with testing frequency (Figure 2). However, even with testing at an average interval of 6 months among those covered by testing, the percentage reductions in prevalence, incidence, and deaths were 45%, 37%, and 42%, respectively, in the sparse network. Deemed infeasible in most settings, testing every 3 months led to reductions of 51%, 42%, and 45%, respectively. The percentage reductions in the dense network setting were equal or slightly smaller.

The cumulative number of treatment initiations did not increase linearly with testing frequency. Testing every 2 years, every year, and every 6 months resulted in annual treatment rates of 31, 38, and 41 per 1000 PWID in the sparse network, respectively; and 40, 52, and 60 per 1000 PWID in the dense network, respectively.

### Sensitivity and Scenario Analyses

**Figure 3.**
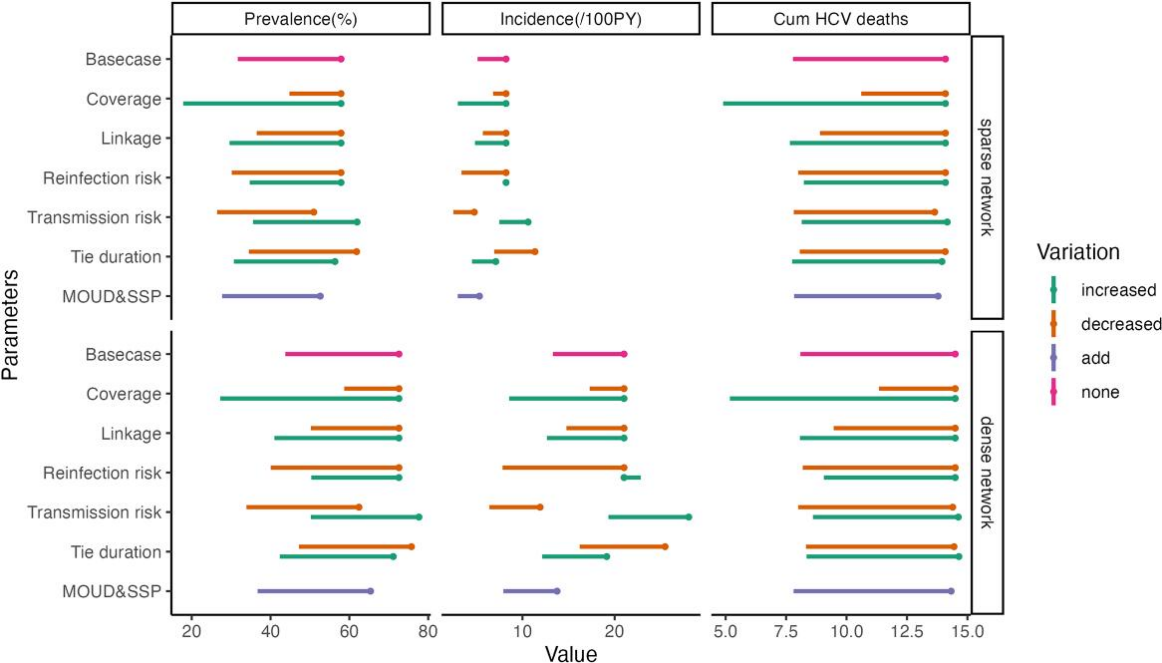
Sensitivity and scenario analysis: impact of parameters governing HCV transmission, testing, and treatment, and ongoing harm-reduction on prevalence, incidence, and cumulative HCV-related deaths over 10 y in PWID. Start point (dot) of each bar is the mean outcome value with no HCV testing, and end point of each bar (the opposite end) is the mean outcome value with testing every 6 months. Each bar shows the absolute change in an outcome from no testing to testing every 6 months (followed by a treatment cascade). Each mean is calculated over 500 iterations of simulations. In each panel, the varied parameter or scenario corresponding to the bars are noted on the *y*-axis: HCV testing coverage, linkage to care rate, relative risk of reinfection compared to primary infection, monthly transmission probability in discordant tie, partnership (tie) duration, and MOUD and SSP intervention in the population. The different colors in a parameter/scenario represent different variations in the parameters or scenarios. Of note, “add” means adding the MOUD and SSP intervention in the population, “none” means no variation in parameters (for the base-case scenario). The *x*-axis shows values of each outcome measure (column title). The top and bottom rows show results for sparse and lower-transmission (average number of injection partners = 1.43) and dense and higher-transmission (average number of injection partners = 3) PWID networks, respectively. The 3 columns show results for 3 outcome measures as abbreviated in the column titles: HCV infection prevalence (%), HCV infection incidence (per 100 PY), and cumulative number of HCV-related deaths over 10 y. Abbreviations: HCV, hepatitis C virus; MOUD, medications for opioid use disorder; PY, person-years; SSP, syringe services program.

Higher levels of testing coverage and linkage to care were associated with increased testing benefits compared to the base case (Figure 3). Testing coverage had a substantial impact: for example, when coverage increased by 50% (from 60% to 90%), testing every six months reduced prevalence, incidence, and HCV-related deaths by 69%, 63%, and 65%, respectively, in the sparse network setting.

Relative risk of reinfection after cure mostly affected incidence reductions. If the reinfection risk was zero, testing every 6 months resulted in relative incidence reductions of 59% in the sparse network. Conversely, if reinfection risk equaled primary infection risk, testing and treatment would marginally increase overall incidence, by 1% and 9% in sparse and dense network settings, respectively (Figure 3).

A lower monthly per-partner transmission probability, an extended duration of injection partnerships, and adding MOUD and SSPs are associated with a decrease in prevalence, incidence, and HCV-related deaths both in the presence and absence of the testing intervention. However, the relative reductions under these conditions were comparable or only slightly greater than those in the base case (Figure 3). For example, testing every six months in the sparse network setting with ongoing MOUD and SSPs led to reductions in prevalence, incidence, and HCV-related deaths by 47%, 44%, and 43%, respectively. When compared to no testing, SSPs, or MOUD (baseline in the base-case), the reductions increased to 52%, 63%, and 44%, respectively (Figure 3). The addition of MOUD and SSPs reduced cumulative deaths attributable to injection drug use from 131 in the base case to 119.

Setting antibody test performance characteristics at values that were less favorable than in the base-case analyses had little effect on the results, reducing the benefits of testing by <3%. Assuming higher spontaneous clearance rates also affected results modestly, by <5% (Supplementary Table 2).

## DISCUSSION

In this study, we evaluated the impact of different HCV testing frequencies on hepatitis C elimination among PWID. We found that increases in testing frequency improved HCV elimination outcomes. However, our results show that testing and treatment alone in the context of the status quo care continuum would not meet the elimination targets. Reductions in a denser network with higher transmission were found to be more difficult, consistent with findings from a previous study suggesting greater treatment rate is needed for settings with higher prevalence [4]. Notably, our base-case scenario assuming 60% coverage, 47% linkage to care, and the absence of explicit harm-reduction interventions reflects ongoing expansion of HCV care and harm-reduction among PWID in the United States, which may vary between locations. Nonetheless, our findings suggest that frequent testing and treatment are necessary; however, they are unlikely to achieve hepatitis C elimination goals in the absence of other interventions and improvements.

Instead of evaluating the treatment rate required to reach elimination goals, which may be less intuitive for practice, we evaluated the impact of increasing average HCV testing frequency in this population. Testing every 6 months yielded an annual treatment rate of 41 per 1000 PWID in the sparse network setting, considerably lower than the treatment need estimated in a previous study of 159 per 1000 PWID required to attain elimination in a comparable population [14]. Our findings also indicate that the cumulative number of treatments did not increase linearly with testing frequency. This is attributable to 2 factors: (1) testing was limited to those who have access to and utilize it and (2) decreases in prevalence associated with increased testing and treatment led to diminishing marginal returns. This highlights the need to adapt strategies to re-identify high-risk individuals.

Our study demonstrates the critical importance of expanding access to and utilization of HCV care (coverage) among PWID. Increasing coverage to 90% could meet the elimination target for HCV-related deaths and significantly reduce incidence, even without other improvements. Many of the barriers to HCV diagnosis and treatment are at the system level, for example, stigma, lack of effective integration of infectious disease prevention and substance use disorder treatment [48]. Innovative, community-engaged models of delivering care are likely essential for reaching this stigmatized and historically underserved population. If a considerable proportion of PWID are not reached by testing and treatment programs, they will remain untreated and continue to spread the virus, even when the remaining PWID engage in frequent testing and treatment [16]. Low-barrier, culturally relevant, and community-based HCV care are crucial to achieve elimination goals.

Our study also emphasizes the importance of preventing reinfection after cure, which had a substantial impact on incidence reduction. Although literature shows that reinfection risk after cure among PWID is lower than primary infection risk, reinfection rates vary considerably in different PWID populations [9, 40]. Our sensitivity analyses show that completely preventing reinfection after cure could increase testing benefits to yield incidence reductions of 59% and 63% in sparse and dense network settings, respectively, representing improvements of 60% to 70% compared to the base-case scenario. Conversely, if DAA treatment and cure have no impact on transmission risk, the benefits of HCV testing and treatment would be substantially reduced, and frequent testing could marginally increase overall incidence due to increased susceptible individuals for reinfection.

Our analysis demonstrates that harm reduction interventions are important components of hepatitis C elimination, as they could contribute significantly to reducing incidence. Notably, deaths attributable to injection drug use were 9 times higher than HCV-related deaths, suggesting that the high competing mortality from drug use may have masked the life-extension benefit of HCV testing and treatment. Furthermore, MOUD and SSPs offer the potential of co-locating HCV services, facilitating accessible and integrated healthcare to PWID, thereby addressing syndemic challenges that disproportionately affect this vulnerable population.

Our study has several limitations. First, the analysis was constrained by the limited availability of dyad-level network data for PWID. Consequently, we leveraged detailed information from 1 study from central Appalachia, combined with review of published literature on rural and urban PWID networks in the United States, to model 1 lower-transmission and 1 higher-transmission setting. The HCV infection prevalence and incidence rates produced by our model align with findings from systematic reviews [39, 49, 50]. Additionally, the comparable outcomes in the 2 settings suggest that our findings are robust to variations in transmission intensity. Second, we did not incorporate specific drug use behaviors such as the types of drugs used or equipment shared, which can further contribute to the heterogeneity in the population. However, we anticipate that their incorporation would not alter our conclusions. Third, we evaluated intervention impacts over a 10-year period; extending the timeframe may yield greater reductions in HCV-related deaths. Finally, although our study provides important evidence on the potential impact of frequent testing toward elimination targets, complementary information on economic outcomes associated with different testing strategies can provide further evidence to inform policymaking and practice.

## CONCLUSION

Although testing PWID for HCV infection as frequently as every 6 months paired with treatment can produce major benefits in reducing HCV incidence, prevalence and mortality, it is unlikely to achieve the hepatitis C elimination goal in the context of the status quo care continuum. Improving access to and utilization of testing among PWID, increasing efforts to prevent reinfection after cure, and complementing testing and treatment with MOUD and SSPs could add substantially to the impact of increased HCV testing and treatment. Substantial extension in life expectancy among PWID likely requires interventions to prevent deaths attributable to overdose and other direct consequences of injection drug use. This requires overcoming structural barriers and improving access to healthcare and prevention services among PWID.

## Supplementary Material

ciaf368_Supplementary_Data

## Supplementary Data

Supplementary materials are available at *Clinical Infectious Diseases* online. Consisting of data provided by the authors to benefit the reader, the posted materials are not copyedited and are the sole responsibility of the authors, so questions or comments should be addressed to the corresponding author.

## Notes

***Author Contributions.*** L. Z., N. W. F., W. W. T., B. P. L., and J. A. S. contributed to design of the study. L. Z. developed the model and conducted the analysis. All authors contributed to interpretation of results. L, Z. wrote the first draft of the manuscript.

***Acknowledgments.*** The authors would like to thank the Statnet team especially Dr Steven Goodreau and Dr Martina Morris for their help and support on using the Statnet packages. The authors would like to acknowledge Dr Jennifer Havens, Dr Abby Rudolph, and Dr April Young for providing the SNAP data and help understanding the data during the development of the static network model that this model is based on, and the participants involved in the SNAP study for sharing their information and experiences, as well as the community-based staff who collected the data. The authors would like to thank Katie Henaghan, Teresa Puente, and Emily Stewart for their management and support during the study.

***Financial support.*** This work was supported by the U.S. Centers for Disease Control and Prevention, National Center for HIV, Viral Hepatitis, STD, and TB Prevention (grant numbers U38PS004644, NU38PS004651) to L. Z., M. B. R., B. P. L., and J. A. S., National Institute on Drug Abuse (grant numbers R37DA015612, K99DA056297) to L. Z., and institutional funding for staff at the Massachusetts Department of Public Health to L. M. R.
