# Supplementary material for: Potential Impact of Frequent Testing for Hepatitis C Virus Among People Who Inject Drugs Toward Hepatitis C Elimination in the United States
Source: Clin Infect Dis. 2025 Jul 15;83(1):e38–46. doi: 10.1093/cid/ciaf368 (PMC13393127; doi:10.1093/cid/ciaf368)
Supplement: ciaf368_Supplementary_Data [file ciaf368_supplementary_data.docx]

**Potential impact of frequent testing for hepatitis C virus among people who inject drugs toward hepatitis C elimination in the United States**

**Supplement**

Lin Zhu, Nathan W. Furukawa, William W. Thompson, Marissa B. Reitsma, Liisa M. Randall, Alice K. Asher, Eduardo Valverde, Benjamin P. Linas, Joshua A. Salomon

**Table S1 Literature review of urban PWID network in the United States**

| **Location** | **Study time** | **Sample size** | **Definition of partner** | **Time counted** | **Mean degree** | **HCV sero prevalence** | **Reference** |
| --- | --- | --- | --- | --- | --- | --- | --- |
| Chicago (young) | 2012-2013 | 164 | Inject most often with | past 6 months | 3.0 | 5.1% | ^[1]^ |
| San Juan | 2012 | 512 | Use needle/works after they injected with it | past 12 months | 2.7/6.3 | 48% | ^[2]^ |
| Chicago, Washington DC | 1995 | 123 | Injection and sexual partner | previous 30 days | 17.4 | NA | ^[3]^ |
| San Francisco | 2005 | 477 | Know other IDU | last 6 months | 18.0-26.0 | 59.5% | ^[4]^ |
| Baltimore (drug user) | 2001-2003 | 742 | Do drugs with | NA | 2.2 | NA | ^[5]^ |
| Baltimore | 1991-1992 | 293 | Do drugs with | last 6 months | 5.2 | NA | ^[6]^ |
| Baltimore | 1993-1994 | 499 | Share drugs | previous 6 months | 3.7 | NA | ^[7]^ |
| Hartford, CT | 2012-2013 | 528 | Inject at the same time and location | previous 6 months | 4.2 | 57.6% | ^[8]^ |

* Considering our definition of partnerships is sharing needles/syringes or other equipment to prepare drugs in the past six months, we selected a mean degree of 3 from these results for our simulation of dense PWID networks.

**Table S2 Impact of hepatitis C virus (HCV) antibody testing performance characteristics and spontaneous clearance rate on relative reductions in HCV infection prevalence, incidence, and HCV-related deaths with different HCV testing frequencies among people who inject drugs (PWID)**

| Parameters | Network | Frequency | Outcome | Mean | SD | Relative change compared to results in main analysis (%) |
| --- | --- | --- | --- | --- | --- | --- |
| Ab sen=96.9% | dense network | every 2 years | cum HCV death | 20 | 27.8 | -0.8 |
| Ab sen=96.9% | dense network | every 2 years | incidence(/100PY) | 21 | 4.4 | -0.4 |
| Ab sen=96.9% | dense network | every 2 years | prevalence(%) | 21 | 1.6 | -0.4 |
| Ab sen=96.9% | dense network | every 6 months | cum HCV death | 40 | 24.2 | -1.6 |
| Ab sen=96.9% | dense network | every 6 months | incidence(/100PY) | 36 | 3.7 | -0.4 |
| Ab sen=96.9% | dense network | every 6 months | prevalence(%) | 39 | 1.7 | -0.4 |
| Ab sen=96.9% | dense network | every year | cum HCV death | 31 | 27.7 | -0.2 |
| Ab sen=96.9% | dense network | every year | incidence(/100PY) | 30 | 4.1 | -0.2 |
| Ab sen=96.9% | dense network | every year | prevalence(%) | 31 | 1.7 | -0.4 |
| Ab sen=96.9% | dense network | none | cum HCV death | 0 | 0.0 | 0.0 |
| Ab sen=96.9% | dense network | none | incidence(/100PY) | 0 | 0.0 | 0.0 |
| Ab sen=96.9% | dense network | none | prevalence(%) | 0 | 0.0 | 0.0 |
| Ab sen=96.9% | sparse network | every 2 years | cum HCV death | 24 | 30.8 | 1.0 |
| Ab sen=96.9% | sparse network | every 2 years | incidence(/100PY) | 21 | 5.9 | -0.5 |
| Ab sen=96.9% | sparse network | every 2 years | prevalence(%) | 26 | 1.9 | -0.6 |
| Ab sen=96.9% | sparse network | every 6 months | cum HCV death | 41 | 24.5 | -1.7 |
| Ab sen=96.9% | sparse network | every 6 months | incidence(/100PY) | 37 | 5.4 | -0.2 |
| Ab sen=96.9% | sparse network | every 6 months | prevalence(%) | 45 | 2.1 | -0.3 |
| Ab sen=96.9% | sparse network | every year | cum HCV death | 36 | 24.8 | 2.1 |
| Ab sen=96.9% | sparse network | every year | incidence(/100PY) | 30 | 5.7 | -0.4 |
| Ab sen=96.9% | sparse network | every year | prevalence(%) | 36 | 2.1 | -0.6 |
| Ab sen=96.9% | sparse network | none | cum HCV death | 0 | 0.0 | 0.0 |
| Ab sen=96.9% | sparse network | none | incidence(/100PY) | 0 | 0.0 | 0.0 |
| Ab sen=96.9% | sparse network | none | prevalence(%) | 0 | 0.0 | 0.0 |
| Ab spe=99% | dense network | every 2 years | cum HCV death | 21 | 27.2 | 0.1 |
| Ab spe=99% | dense network | every 2 years | incidence(/100PY) | 22 | 4.5 | 0.4 |
| Ab spe=99% | dense network | every 2 years | prevalence(%) | 21 | 1.6 | 0.2 |
| Ab spe=99% | dense network | every 6 months | cum HCV death | 39 | 24.1 | -2.5 |
| Ab spe=99% | dense network | every 6 months | incidence(/100PY) | 37 | 3.5 | 0.4 |
| Ab spe=99% | dense network | every 6 months | prevalence(%) | 40 | 1.6 | 0.3 |
| Ab spe=99% | dense network | every year | cum HCV death | 32 | 26.4 | 0.5 |
| Ab spe=99% | dense network | every year | incidence(/100PY) | 30 | 3.9 | 0.0 |
| Ab spe=99% | dense network | every year | prevalence(%) | 31 | 1.6 | 0.0 |
| Ab spe=99% | dense network | none | cum HCV death | 0 | 0.0 | 0.0 |
| Ab spe=99% | dense network | none | incidence(/100PY) | 0 | 0.0 | 0.0 |
| Ab spe=99% | dense network | none | prevalence(%) | 0 | 0.0 | 0.0 |
| Ab spe=99% | sparse network | every 2 years | cum HCV death | 25 | 29.3 | 2.3 |
| Ab spe=99% | sparse network | every 2 years | incidence(/100PY) | 21 | 6.0 | -0.8 |
| Ab spe=99% | sparse network | every 2 years | prevalence(%) | 26 | 2.0 | -0.3 |
| Ab spe=99% | sparse network | every 6 months | cum HCV death | 40 | 25.7 | -1.9 |
| Ab spe=99% | sparse network | every 6 months | incidence(/100PY) | 37 | 5.4 | 0.0 |
| Ab spe=99% | sparse network | every 6 months | prevalence(%) | 45 | 2.1 | 0.0 |
| Ab spe=99% | sparse network | every year | cum HCV death | 35 | 28.6 | 1.0 |
| Ab spe=99% | sparse network | every year | incidence(/100PY) | 30 | 5.7 | 0.0 |
| Ab spe=99% | sparse network | every year | prevalence(%) | 37 | 2.1 | -0.1 |
| Ab spe=99% | sparse network | none | cum HCV death | 0 | 0.0 | 0.0 |
| Ab spe=99% | sparse network | none | incidence(/100PY) | 0 | 0.0 | 0.0 |
| Ab spe=99% | sparse network | none | prevalence(%) | 0 | 0.0 | 0.0 |
| spontaneous clearance=50% | dense network | every 2 years | cum HCV death | 18 | 38.2 | -3.1 |
| spontaneous clearance=50% | dense network | every 2 years | incidence(/100PY) | 25 | 4.1 | 3.3 |
| spontaneous clearance=50% | dense network | every 2 years | prevalence(%) | 24 | 1.7 | 3.3 |
| spontaneous clearance=50% | dense network | every 6 months | cum HCV death | 41 | 28.0 | -1.0 |
| spontaneous clearance=50% | dense network | every 6 months | incidence(/100PY) | 41 | 3.6 | 4.8 |
| spontaneous clearance=50% | dense network | every 6 months | prevalence(%) | 43 | 1.9 | 3.8 |
| spontaneous clearance=50% | dense network | every year | cum HCV death | 32 | 36.2 | 0.5 |
| spontaneous clearance=50% | dense network | every year | incidence(/100PY) | 34 | 3.7 | 4.1 |
| spontaneous clearance=50% | dense network | every year | prevalence(%) | 35 | 1.8 | 3.8 |
| spontaneous clearance=50% | dense network | none | cum HCV death | 0 | 0.0 | 0.0 |
| spontaneous clearance=50% | dense network | none | incidence(/100PY) | 0 | 0.0 | 0.0 |
| spontaneous clearance=50% | dense network | none | prevalence(%) | 0 | 0.0 | 0.0 |
| spontaneous clearance=50% | sparse network | every 2 years | cum HCV death | 22 | 38.3 | -0.9 |
| spontaneous clearance=50% | sparse network | every 2 years | incidence(/100PY) | 26 | 5.5 | 3.7 |
| spontaneous clearance=50% | sparse network | every 2 years | prevalence(%) | 28 | 1.8 | 1.9 |
| spontaneous clearance=50% | sparse network | every 6 months | cum HCV death | 40 | 33.7 | -2.2 |
| spontaneous clearance=50% | sparse network | every 6 months | incidence(/100PY) | 43 | 5.2 | 5.5 |
| spontaneous clearance=50% | sparse network | every 6 months | prevalence(%) | 47 | 2.1 | 2.0 |
| spontaneous clearance=50% | sparse network | every year | cum HCV death | 30 | 38.7 | -3.9 |
| spontaneous clearance=50% | sparse network | every year | incidence(/100PY) | 36 | 5.3 | 5.3 |
| spontaneous clearance=50% | sparse network | every year | prevalence(%) | 39 | 2.0 | 2.2 |
| spontaneous clearance=50% | sparse network | none | cum HCV death | 0 | 0.0 | 0.0 |
| spontaneous clearance=50% | sparse network | none | incidence(/100PY) | 0 | 0.0 | 0.0 |
| spontaneous clearance=50% | sparse network | none | prevalence(%) | 0 | 0.0 | 0.0 |

**Technical Appendix**

Table of Contents

[1. Age distributions 10](#_Toc191125051)

[1.1 Initial network and migrated individuals 10](#_Toc191125052)

[1.2 New injectors 10](#_Toc191125053)

[1.3 Aging 10](#_Toc191125054)

[2. Fibrosis distribution 11](#_Toc191125055)

[3. Initiation, cessation, and relapse of injection drug use 12](#_Toc191125056)

[3.1 Initiation 12](#_Toc191125057)

[3.2 Cessation and relapse 12](#_Toc191125058)

[4. Mortality 13](#_Toc191125059)

[4.1 Age dependent base mortality 13](#_Toc191125060)

[4.2 Age-dependent drug-use-related standardized mortality ratio (SMR) 13](#_Toc191125061)

[4.3 Excess mortality due to F4 and decompensation 13](#_Toc191125062)

[4.4 Impact of sustained virologic response (SVR) on excess mortality due to F4 or decompensation 14](#_Toc191125063)

[5. HCV antibody development after acute infection 15](#_Toc191125064)

[References 16](#_Toc191125065)

# 1. Age distributions

## 1.1 Initial network and migrated individuals

We fitted normal, lognormal, gamma and Weibull distributions to the age pattern observed in the SNAP PWID sample and selected the lognormal distributions as the best fitting result based on AIC and BIC values.


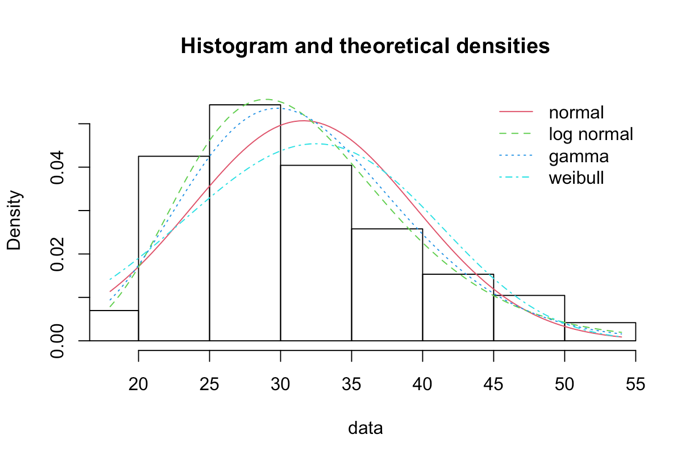

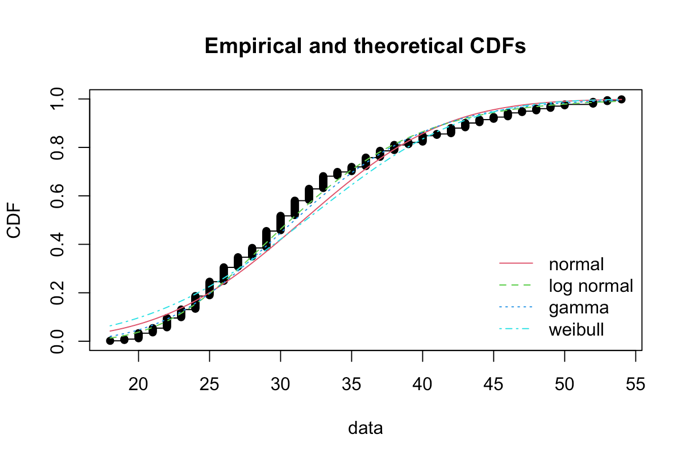


We used the fitted distribution (mean log=3.424, sd log=0.241) truncating at the minimum age of injection initiation = 13 to assign age distributions to the initial network and to individuals who migrate into the network.

## 1.2 New injectors

We identified the best-fitting distribution to the observed distribution of ages at injection initiation among the SNAP PWID following the same approach as above, with the lognormal distribution producing the best fitting results.


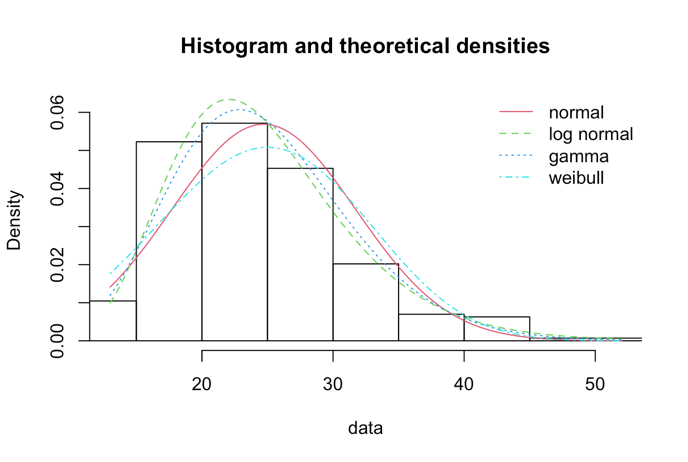

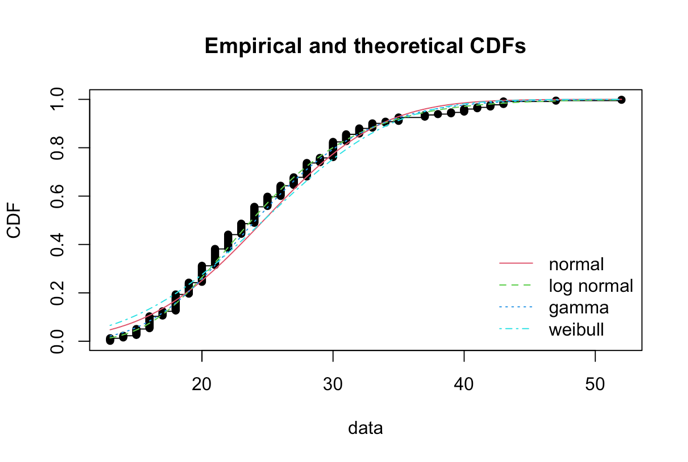


We used the fitted distribution (mean log=3.169, sd log=0.275) truncating at the minimum age of injection initiation = 13 to assign the age distribution for new injectors.

## 1.3 Aging

We updated age for all simulated individuals in the model at yearly intervals.

# 2. Fibrosis distribution

We assigned F0 to individuals who have never been infected. For individuals with current or former infection, we gathered numbers of patients in different fibrosis stages (none/mild, moderate, and advanced/cirrhosis) by birth cohort (born before 1945, 1945-1965, and after 1965) and HCV infection status (currently infected and resolved/cured) from Table 1 in the Klevens et al. study. ^[9]^ We converted the numbers of patients to percentages, and mapped none/mild to F0/F1, moderate to F1/F2, and advanced/cirrhosis to F3/F4/decompensated according to matching of FIB-4, Ishak 6, and METAVIR (FIB-4 maps to Ishak 0-1, 2-3, 4-6; Ishak 0-1 maps to METAVIR F0/F1; Ishak 2-3 maps to METAVIR F1/F2; and Ishak 4-6 maps to F3/F4). We implemented 50/50 splits between F0 and F1, F1 and F2, and 50/40/10 split between F3, F4, and decompensated. The table below shows results of these calculations.

Table Fibrosis distribution among individuals with current or former HCV infection

| Age | Infection Status | F0 | F1 | F2 | F3 | F4 | Decomp |
| --- | --- | --- | --- | --- | --- | --- | --- |
| 65-100 | Current | 0.0372 | 0.2665 | 0.2293 | 0.2335 | 0.1868 | 0.0467 |
| 65-100 | Resolved/Cured | 0.1423 | 0.4221 | 0.2798 | 0.0779 | 0.0623 | 0.0156 |
| 45-64 | Current | 0.1465 | 0.3641 | 0.2176 | 0.1359 | 0.1087 | 0.0272 |
| 45-64 | Resolved/Cured | 0.3303 | 0.4693 | 0.1390 | 0.0307 | 0.0245 | 0.0061 |
| 0-44 | Current | 0.3981 | 0.4740 | 0.0759 | 0.0260 | 0.0208 | 0.0052 |
| 0-44 | Resolved/Cured | 0.4713 | 0.4946 | 0.0233 | 0.0054 | 0.0043 | 0.0011 |

# 3. Initiation, cessation, and relapse of injection drug use

## 3.1 Initiation

We calibrated the initiation rate to make the size of the active injection network stable (around 1000 current PWID) in the status quo (no intervention) scenario. We assumed that each person is HCV naïve at injection drug use initiation.^[10]^

## 3.2 Cessation and relapse

We estimated monthly rates of injecting cessation and injecting relapse, and the probability of permanent cessation, from the ALIVE cohort study conducted in Baltimore, MD over 1988 to 2000. ^[11]^ The following steps were used:

1. We digitized the time to cessation and relapse curves reported by Shah NG et al. ^[11]^ in Figure 1 (Kaplan–Meier of observed time from baseline to first cessation of injection, and time to first relapse to injection following cessation), extracting 25 data points from each curve.
2. We the asymptote of the relapse curve to estimate the probability of permanent cessation, which was approximately 13%.
3. We fitted an exponential function to the digitized data points of the cessation curve with a forced intercept of 100. The exponential function was estimated as Y = 100 exp (-0.168 X), with an adjusted R-squared of 0.9988. Based on the estimated coefficient, we computed the monthly cessation rate to be 0.168/12 = 0.014.
4. For the relapse curve, we first converted the Y values to be conditional on ever relapsing (i.e., allowing for the curve to approach an asymptote at 13%): Z = (Y-13)/(100-13)×100. Then we fitted an exponential function using the same method as the cessation curve, and the result was Z = 100 exp (-0.454 X), with an adjusted R-squared of 0.9589. We computed the monthly relapse rate as 0.454/100×(100-13)/12 = 0.033.

PWID who cease injection are characterized as “former PWID” and pause their injection partnerships. When former PWID relapse, they return to the active PWID network and re-acquire previous partners if they remain in the network. PWID who initiate injection are assigned partnerships to preserve network mean degree; partnerships of PWID who die are removed permanently.

# 4. Mortality

## 4.1 Age dependent base mortality

We used 2019 United States life tables.^[12]^ We downloaded life tables for males (https://ftp.cdc.gov/pub/Health_Statistics/NCHS/Publications/NVSR/70-19/Table02.xlsx) and females (https://ftp.cdc.gov/pub/Health_Statistics/NCHS/Publications/NVSR/70-19/Table02.xlsx) on May 1 2023. We converted mortality probabilities to rates, calculated both-sexes rates using the sex distribution reported for the SNAP PWID sample (59% male), and converted yearly rates back to monthly probabilities. The probability of death at age 100 was set to 1.

## 4.2 Age-dependent drug-use-related standardized mortality ratio (SMR)

We identified SMRs associated with drug use from the Evans et al.^[13]^ cohort study among individuals accessing pharmacological treatment for opioid dependence, which reported point estimates of 1.8 and 6.1 for SMRs among former and current drug use, respectively, and an overall estimate of 4.5. The study also showed that the SMR decreased by age (Table 3 in the publication), consistent with results of a systematic review ^[14]^ and analysis of the ALIVE study. ^[15]^ Based on these findings, we estimated age-dependent drug-use-related SMRs using the following calculations.

We calculated the ratios of current or former drug use SMRs to SMRs in the overall cohort with an offset of 1:

$$\mathrm{rati}o_{\mathrm{current}}=\frac{current SMR-1}{overall SMR-1}=\frac{6.1-1}{4.5-1}=1.457$$

$$\mathrm{rati}o_{\mathrm{former}}=\frac{former SMR-1}{overall SMR-1}=\frac{1.8-1}{4.5-1}=0.229$$

We fitted a regression to reported age-specific SMRs in Evans et al., which resulted in the estimated function log(SMR-1) = 2.6193 – 0.0304×Age. We used this estimated regression to generate overall SMRs by age, and then used the two ratios calculated in the first step to estimate SMRs by age for current and former injectors.

## 4.3 Excess mortality due to F4 and decompensation

We calculated excess mortality for compensated and decompensated cirrhosis from results reported in a cohort study by Bruno et al.^[16]^ as follows:

1. From numbers in Figure 1 in the report, the number of liver deaths and annual rate among untreated patients were 60 and 3/100 person-years (py), respectively, implying total person-time of 60/0.03=2000 py; the reported number of liver deaths and annual rate among treated patients without sustained virological response (SVR) were 46 and 2.4/100 py, respectively, implying total person-time is 46/0.024=1917 py.
2. We combined decompensation and HCC in our model as one group representing severe liver disease, so our calculation from this study was also based on this grouping.
3. From Figure 3 in the report, the number of deaths with all combinations of decompensation and HCC from untreated and treated patients without SVR was 30+10+15+47=102, and total person-years was 301+31+251+80=663.
4. Combining numbers in steps a and c, the number of deaths for F4 was 60+46-102=4, person-time was 2000+1917-663=3254, and the annual rate was 4/3254=0.12/100 py, implying a monthly excess mortality rate due to F4 of 0.012/100/12=0.0001.
5. Similarly, the number of deaths for decompensated cirrhosis (plus HCC) was 102/663=15.38/100 py, implying a monthly excess mortality rate due to decompensation (plus HCC) of 0.0128.

## 4.4 Impact of sustained virologic response (SVR) on excess mortality due to F4 or decompensation

Based on results of an international, multicenter, long-term follow-up study by van der Meer et al. (Table 4 in the report), ^[17]^ we multiplied the hazard ratio (HR) for liver-related mortality by SVR and Fibrosis Ishak 6 (corresponding to F4, and we assume similar HR for decompensation) 0.06×4.84=0.29 to derive the HR for liver-related mortality following SVR among F4 and decompensation patients.

# 5. HCV antibody development after acute infection

Antibodies to HCV typically become detectable within 20 to 150 days after infection (mean 60 days), ^[18]^ so we assumed 5% becoming detectable within one month after infection, and 50% within two months after infection. After 12 weeks, more than 90% of patients will have a positive HCV antibody test, ^[19]^ so we assumed 90% within three months after infection. In rare cases, there are individuals who do not develop antibody, and we assumed this percentage to be 1%. ^[20]^

# References

1. Boodram B, Hotton AL, Shekhtman L, Gutfraind A, Dahari H. High-risk geographic mobility patterns among young urban and suburban persons who inject drugs and their injection network members. Journal of Urban Health **2018**; 95(1): 71-82.

2. Thrash C, Welch-Lazoritz M, Gauthier G, et al. Rural and urban injection drug use in Puerto Rico: Network implications for human immunodeficiency virus and hepatitis C virus infection. Journal of ethnicity in substance abuse **2018**; 17(2): 199-222.

3. Hoffmann JP, Su SS, Pach A. Changes in network characteristics and HIV risk behavior among injection drug users. Drug and alcohol dependence **1997**; 46(1-2): 41-51.

4. Malekinejad M, McFarland W, Vaudrey J, Raymond HF. Accessing a diverse sample of injection drug users in San Francisco through respondent-driven sampling. Drug and alcohol dependence **2011**; 118(2-3): 83-91.

5. Latkin CA, Hua W, Tobin K. Social network correlates of self-reported non-fatal overdose. Drug and Alcohol Dependence **2004**; 73(1): 61-7.

6. Latkin C, Mandell W, Oziemkowska M, et al. Using social network analysis to study patterns of drug use among urban drug users at high risk for HIV/AIDS. Drug and alcohol dependence **1995**; 38(1): 1-9.

7. Suh T, Mandell W, Latkin C, Kim J. Social network characteristics and injecting HIV-risk behaviors among street injection drug users. Drug and alcohol dependence **1997**; 47(2): 137-43.

8. Zelenev A, Li J, Mazhnaya A, Basu S, Altice FL. Hepatitis C virus treatment as prevention in an extended network of people who inject drugs in the USA: a modelling study. The Lancet Infectious Diseases **2017**.

9. Klevens RM, Canary L, Huang X, et al. The burden of hepatitis C infection–related liver fibrosis in the United States. Clinical Infectious Diseases **2016**; 63(8): 1049-55.

10. Fuller CM, Ompad DC, Galea S, Wu Y, Koblin B, Vlahov D. Hepatitis C incidence—a comparison between injection and noninjection drug users in New York City. Journal of Urban Health **2004**; 81(1): 20-4.

11. Shah NG, Galai N, Celentano DD, Vlahov D, Strathdee SA. Longitudinal predictors of injection cessation and subsequent relapse among a cohort of injection drug users in Baltimore, MD, 1988–2000. Drug and alcohol dependence **2006**; 83(2): 147-56.

12. Arias E, Xu J, Tejada-Vera B, Bastian B. United States life tables, 2019. **2022**.

13. Evans E, Li L, Min J, et al. Mortality among individuals accessing pharmacological treatment for opioid dependence in California, 2006–10. Addiction **2015**; 110(6): 996-1005.

14. Larney S, Tran LT, Leung J, et al. All-cause and cause-specific mortality among people using extramedical opioids: a systematic review and meta-analysis. JAMA psychiatry **2020**; 77(5): 493-502.

15. Cepeda JA, Astemborski J, Kirk GD, Celentano DD, Thomas DL, Mehta SH. Rising role of prescription drugs as a portal to injection drug use and associated mortality in Baltimore, Maryland. PLoS One **2019**; 14(3): e0213357.

16. Bruno S, Zuin M, Crosignani A, et al. Predicting Mortality Risk in Patients With Compensated HCV-lnduced Cirrhosis: A Long-Term Prospective Study. Official journal of the American College of Gastroenterology| ACG **2009**; 104(5): 1147-58.

17. van der Meer AJ, Veldt BJ, Feld JJ, et al. Association between sustained virological response and all-cause mortality among patients with chronic hepatitis C and advanced hepatic fibrosis. Jama **2012**; 308(24): 2584-93.

18. Busch MP, Page Shafer KA. Acute-phase hepatitis C virus infection: implications for research, diagnosis, and treatment. Vol. 40: The University of Chicago Press, **2005**:959-61.

19. Rena K. Fox MAC. Diagnosis of Acute HCV Infection. Available at: <https://www.hepatitisc.uw.edu/go/screening-diagnosis/acute-diagnosis/core-concept/all>.

20. Stramer SL, Glynn SA, Kleinman SH, et al. Detection of HIV-1 and HCV infections among antibody-negative blood donors by nucleic acid–amplification testing. New England Journal of Medicine **2004**; 351(8): 760-8.
